# Efficient MW-Assisted Synthesis, Spectroscopic Characterization, X-ray and Antioxidant Properties of Indazole Derivatives

**DOI:** 10.3390/molecules21070903

**Published:** 2016-07-09

**Authors:** Efrain Polo, Jorge Trilleras, Juan Ramos, Antonio Galdámez, Jairo Quiroga, Margarita Gutierrez

**Affiliations:** 1 Organic Synthesis Laboratory and Biological Activity (LSO-Act-Bio), Institute of Chemistry of Natural Resources, Universidad de Talca, Casilla 747, Talca 3460000, Chile; epolo@utalca.cl; 2 Grupo/Semillero de Investigación en Compuestos Heterocíclicos, Programa de Química, Facultad de Ciencias Básicas, Universidad del Atlántico, Km 7 Antigua vía Puerto Colombia, Barranquilla 081007, Atlántico, Colombia; jorgetrilleras@mail.uniatlantico.edu.co (J.T.); jotaramos87@gmail.com (J.R.); 3 Solid State Chemistry Laboratory, Science Faculty, University of Chile, Santiago, 7800003, Chile; agaldamez@uchile.cl; 4 Heterocyclic Compounds Research Group, Department of Chemistry, Universidad del Valle, Cali A.A. 25360, Colombia; jairo.quiroga@correounivalle.edu.co

**Keywords:** tetrahydroindazole, antioxidant activity, microwave irradiation, green chemistry

## Abstract

A small series of tetrahydroindazoles was prepared, starting from 2-acetylcyclohexanone and different hydrazines using reflux and a focused microwave reactor. Microwave irradiation (MW) favored the formation of the desired products with improved yields and shortened reaction times. This is a simple and green method for the synthesis of substituted tetrahydroindazole derivatives. The in vitro antioxidant activity was evaluated using the DPPH and ABTS methods. In these assays, 2-(4-fluorophenyl)-3-methyl-4,5,6,7-tetrahydro-2*H*-indazole (**3f**) showed moderate DPPH decoloring activity, while 3-methyl-4,5,6,7-tetrahydro-1*H*-indazole (**3a**), 3-methyl-2-phenyl-4,5,6,7-tetrahydro-2*H*-indazole (**3b**) and 2-(4-fluorophenyl)-3-methyl-4,5,6,7-tetrahydro-2*H*-indazole (**3f**) were the most active in the ABTS assay. All compounds were well characterized by IR, ^1^H-, ^13^C-NMR and GC-MS spectroscopy and physical data, while the structure of 4-(3-methyl-4,5,6,7-tetrahydro-2*H*-indazol-2-yl)benzoic acid (**3e**) was also determined by single crystal X-ray analysis.

## 1. Introduction

In the last decade there has been rapid progress in the synthetic organic chemistry field associated with the search for new organic compound derivatives with desirable properties. Such compounds are widely used in the pharmaceutical industry. The search for new synthetic strategies that enable higher performance, less byproducts and shorter reaction times also seeks new non-conventional energy sources for obtaining products with industrial, pharmaceutical or other applications. Microwave-assisted synthesis is a branch of green chemistry that has gained much attention in recent years. Moreover, it is considered pollution-free and eco-friendly and typically offers high yields, together with simplified processing and handling [1] as compared to conventional synthesis methods. Its use in organic synthesis has dramatically increased in the last few years, receiving widespread acceptance and becoming an indispensable tool for obtain new structures and it is recognized as a valuable tool for easing some of the bottlenecks in the drug discovery process [2,3,4,5]. In this method, reactions occur more rapidly, safely, and with higher chemical yields, often far better than conventional methods, which require longer reaction times and larger quantities of solvents and reagents, cause environmental pollution, and contribute to health hazards [6]. Many heterocyclic compounds have been synthetized using MW irradiation [7] and Katritzky and Singh reported the applications of MW technology in well-known cyclisation reactions for heterocyclic ring formation and on other important reactions, such as nucleophilic substitution, hetero-Diels-Alder reactions, 1,3-dipolar cycloaddition [8]. Six-membered heterocycles and their fused derivatives has been reported among the compounds synthetized via MW irradiation, using cyclocondensation, cycloaddition, multicomponents and other modular reactions, generating good yield and short reaction time in an easy and rapid way [9]. Tetrahydroindazole synthesis using MW irradiation has been previously reported [10,11,12].

Tetrahydroindazole are chemical compounds of synthetic origin with a five-membered heterocycle, two nitrogen atoms, and three adjacent carbons. Tetrahydroindazole are potent medicinal scaffolds and exhibt a full spectrum of biological activities [13,14,15,16,17]. Due to their attractive pharmacological properties tetrahydroindazole derivatives have attracted the attention of chemists who have researched ways to obtain the desired properties through different synthetic strategies [18]. The most common reactions include: (i) reaction of 2-substituted cyclohexanone with a hydrazine; (ii) ring closure of o-chlorocyclohexanonephenylhydrazone; (iii) reaction of 2-chlorocyclohexenal with hydrazine hydrate; (iv) addition of diazomethane to cyclohexene; (v) hydrogenation of indazoles with platinum in acetic acid; and (vi) the hydrolysis of 1-carbamyl-4,5,6,7-tetrahydroindazole [19]. Accordingly, tetrahydroindazole synthesis has long been the subject of interest. At the beginning of the 20th century, tetrahydroindazole was first prepared by Ainsworth, the reaction of 2-hydroxymethylenecyclohexanone with hydrazine hydrate in methyl alcohol [20]. Since then, Ainsworth´s tetrahydroindazole synthesis has been adopted as the standard method because of its convenience and versatility.

Based on the careful analysis of the literature, the present investigation was aimed to focus on the synthesis of pyrazoles using conventional and non-conventional methods. The tetrahydroindazole series was synthesized by conventional methods, using different solvents, and MW irradiation methods with the purpose of comparing purity, yield and reaction time. The isolated and purified compounds were characterized on the basis of IR, ^1^H-, ^13^C-NMR and mass spectral data. The antioxidant activity was evaluated using the DPPH and ABTS methods. In this context, we present an efficient, clean and straightforward procedure to prepare a series of tetrahydroindazole derivatives by MW irradiation under solvent-free conditions, with short reaction times, and using ethanol as a green solvent in order to obtain the tetrahydroindazole derivatives from crude reaction mixtures without the use of complicated work-up.

## 2. Results and Discussions

**Scheme 1:**
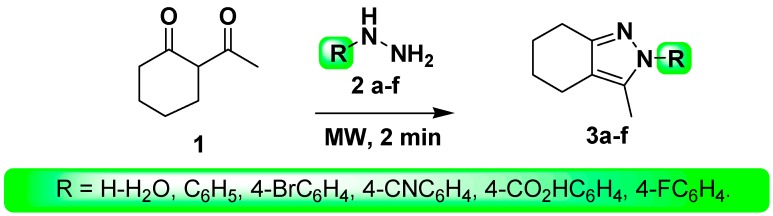
Synthesis of compounds **3a**–**f**.

Six tetrahydroindazole derivatives with different patterns of substitution were synthesized using a modified Paal-Knorr reaction [21] between 1,3-dicarbonyl compounds and hydrazines (Scheme 1). This resulted in good yields with almost no secondary products. These coupling reactions were performed under reflux and MW irradiation, that helped obtain the best results. Reaction conditions, substituents, yields and reaction times are listed in Table 1.

With the results obtained it can be established that the state of aggregation of the reagents is not a limiting factor for the use of microwaves, since in this work, both liquid and solid reagents were used. Reagent **1** was used in a liquid state such as the hydrazine to obtain **3a** and **3b**; the hydrazines used to obtain the compounds **3c**–**f** were solids. This feature is consistent with the principles of green chemistry recommending a decrease in the use of solvents. The compounds **3a** and 3**b** were obtained as oils (Table 1).

Dimethylformamide and acetic acid were used in conventional method with the purpose of comparing the influence of solvent on yield and reaction time. Acetic acid is one of the most important chemical intermediates and frequently used aliphatic carboxylic acids. Acetic and some other acids and their alkali-metal salts are components of buffers for pH measurements in mixed solvents. DMF as a solvent with moderately high permittivity and aprotic nature is useful for acid-base studies and for a wide range of organic reactions. It appears to be a suitable co-solvent since it is aprotic and fully miscible with water [22].

This comparison in the use of a polar protic solvent (acetic acid) and another aprotic apolar one (DMF) allowed us to establish that although there is a decrease in reaction times by using acetic acid, the reaction yield was not favored, as the use of DMF led to an increase in reaction yields for all compounds. This may be related to the characteristics of aprotic solvents which are capable of accepting hydrogen bonds, do not have acidic hydrogens and are capable of dissolving salts, which facilitates the formation of tetrahydroindazoles, in contrast, the use of acetic acid, a protic polar solvent, is characterized by display hydrogen bonding, have an acidic hydrogen and dissolve salts. Based on the dielectric constants of the solvents used, it is confirmed that solvents with high dielectric constant values are favorable for nucleophilic attack on carbonyl groups.

Comparative analyses of percentage yields and total reaction time for all synthesised tetrahydroindazole derivatives by both conventional and MW-assisted methods were performed to determine if MW-assisted synthesis of tetrahydroindazole derivatives added any advantage (Table 1). It was found that yields were improved while total reaction times were reduced drastically. This would be highly advantageous for drug discovery laboratories where small amounts of different analogues have to be synthesized in short periods of time, as well as combinatorial synthesis of new libraries of compounds [23].

Optimal working conditions were determined using microwave for synthesis, varying the temperature and reaction time as shown in Table 2. For example, for compound **3c**, for which it was found that 150 °C and 2 min were the best conditions, keeping the power constant at 300 W.

Compounds **3c**–**f** were purified by precipitation and were obtained as solids, while compounds **3a** and **3b** were obtained as oils. The structures and formulas of all the synthesized compounds were supported by physical and spectral data and found to be in good agreement with the target compounds. Tetrahydroindazoles **3a**–**f** were characterized by ^1^H-NMR, ^13^C-NMR and mass spectroscopy. The ^1^H-NMR spectra of the tetrahydroindazole derivatives were very similar, and characterized by the presence of three groups of signals indicating the presence of aromatic protons, protons near heteroatoms and aliphatic protons, which resonated in the different regions.

The use of MW irradiation for carrying out chemical transformations produces eco-friendly processes. The basis of this technique in organic synthesis is the empirical observation that some reactions proceed much faster and with higher yields compared to conventional heating. MW irradiation facilitates the polarization of the molecules under irradiation. This causes an increase in the reaction rate, which is reflected in significant reduction in reaction times ranging from hours to minutes. The use of MW radiation for the synthesis of tetrahydroindazoles has previously been reported [24] showing increases in yields and significant decrease in reaction times. With the decrease in the use of solvents, it was possible to obtain more consistent products in line with the principles of green chemistry.

**Figure 1:**
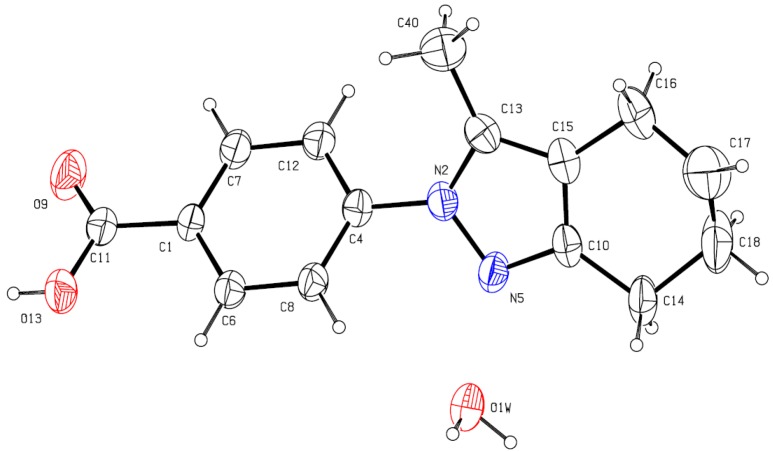
A view of molecular structure showing the displacement ellipsoids are drawn at the 50% probability level. H-atoms are shown as small spheres of arbitrary radii.

Compound **3e** was isolated as a single-crystal, and Figure 1 shows the molecular structure with the atom numbering scheme and a summary of crystallographic data for the compound is given in Table 3. The torsion angle of C13-N2-C4-C12 is 43.7 (5)°. The Cremer and Pople puckering parameters of 6-membered ring (C10/C14/C18-C15) are: Q2 is 0.359 (6) Å, θ is 49.0 (8)° and φ is 148.4 (11)°. The torsion angle (C7-C1-C1-O9) at carboxyl substituent group is 9.2 (6)°. This group is almost coplanar with aromatic ring. All relevant structural parameters (bond distances and angles) are as expected and in acceptable agreement with other organic molecules [25]. Crystallographic data reported in this paper for compound **3e** has been deposited at the Cambridge Crystallographic Data Center (CCDC), No. 1470060. Copies of the data can be obtained, free of charge, on application to CCDC 12 Union Road, Cambridge CB2 1EZ, UK (Fax: +44-1223-336033 or e-mail: deposit@ccdc.cam.ac.uk).

**Figure 2:**
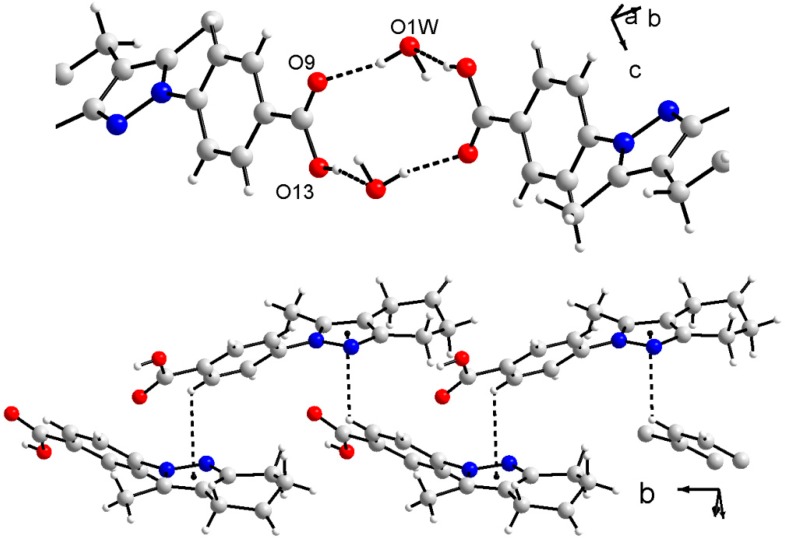
A view of partial packing of **3e** showing: (top) the central core linking the components mediated by hydrogen bonds and (bottom) C-H...π (tetrahydroindazole) interactions. H-atoms are shown as small spheres of arbitrary radii. Dashed lines represent intermolecular interactions.

The supramolecular architecture is assembled from the molecules with solvent water molecules *via* hydrogen-bonding interactions. The water molecules serve to link molecules forming two-dimensional array (Figure 2). Each water molecule acts as an acceptor, in O-H···OW hydrogen bond, and as a donor. The O1W-H2W···O9 distance is 1.91 Å (angle of 166°) and O13-H13···O1W distance is 1.78 Å (angle of 166°). The central core constitutes a graph-set descriptor $R_{4}^{4}(12)$ motif (Figure 2-top) [26]. The network is reinforced by C-H···π (pyrazole) interactions [27]. The C7-H7···*Cg1* distance is 2.90 Å and Cg1[symmetry code: 1/2 − x, 1/2 + y, 3/2 − z] represents the centroid of the tetrahydroindazole ring. These intermolecular interaction form chains run zig-zagging parallel to the [010] plane (Figure 2-top).

In using natural sources as the basis to develop new drugs, industry usually faces the challenge of low concentrations of selected substances that generally make their commercial exploitation unfeasible. However, the synthesis of these substances and their derivatives frequently allow the pharmacophore to be established, and modulation of biological profiles represent an excellent opportunity to explore the biological actions of these synthetic and semi-synthetic compounds [28].

Antioxidant activity is related to compounds capable of protecting a biological system against the harmful effects of oxidative stress. Antioxidants have received increased attention in recent years from nutritionists and medical researchers for their potential activities in the prevention of several degenerative diseases such as cancer and cardiovascular disorder, as well as aging [29]. On the other hand, there are few reports on the antioxidant activities of synthetic compounds [30]. Consequently the synthesis of new active derivatives with potential applications in this area and prepared by simple chemical procedures are of increasing interest. In order to have a standard antioxidant activity measurement system to compare with natural antioxidants and to be incorporated into food, synthetic antioxidants have been developed. Synthetic antioxidants compounds are widely used in food products to inhibit progress of lipid oxidation [31]. In the United States, the following synthetic antioxidants have been evaluated by the FDA and are the most widely used: *tert*-butylhydroquinone (TBHQ), butylated hydroxyanisole (BHA), butylated hydroxytoluene (BHT) and propyl gallate [32]. These antioxidants are cheaper and easier to process than natural antioxidants. Used in approved quantities, synthetic antioxidants are safe and effective [33]. Synthetic tetrahydroindazoles and derivatives have been reported as good antioxidants [34,35,36,37], therefore the research and evaluation of tetrahydroindazole derivatives are a promising area of study for obtained new compounds to use as antioxidant agents.

All compounds obtained were evaluated as antioxidant agents. As shown in Table 4, the DPPH scavenging activity of the compounds varied greatly. The data showed clearly that compound **3f** has good activity in both assays, with best activity in the capture assay ABTS radical, showing better IC_50_ value than ascorbic acid used as a reference (Table 5). Obtained results are concordant with results previously reported where described compounds having electron with- drawing groups like chlorine and fluorine, and electron donating groups like methoxy on the phenyl rings attached to tetrahydroindazole exhibit good antioxidant activity [38].

## 3. Materials and Methods

### 3.1. General Information

The experiments were performed in a Discover microwave apparatus (CEM Corporation, Matthews, NC, USA). All the products were characterized by spectral data (IR, MS, ^1^H-NMR, ^13^C-NMR). ^1^H and ^13^C-NMR spectra (400.1 MHz for proton and 100.6 MHz for carbon) were recorded on an AM-400 spectrometer (Bruker, Rheinstetten, Germany), using CDCl_3_, DMSO-*d_6_* and CD_3_OD as solvents. Tetramethylsilane (TMS) was used as an internal standard. Chemical shifts (δ) and *J* values are reported in ppm and Hz, respectively, relative to the solvent peak CDCl_3_ at 7.24 ppm for protons and 77 ppm for carbon atoms; DMSO-*d*_6_ 2.5 ppm for protons and 39.7 ppm for carbon atoms, and CD_3_OD with 3.35 and 4.78 ppm for protons and 49.3 ppm for carbon. Signals are designated as follows: s, singlet; d, doublet; dd, doublet of doublets; t, triplet; m, multiplet; br.s, broad singlet. IR spectra (KBr pellets, 500–4000 cm^−1^) were recorded on a NEXUS 670 FT-IR spectrophotometer (Thermo Nicolet, Madison, WI, USA). GC-MS analyses were performed on a model Trace 1300 GC-MS instrument (Thermo Fisher Scientific, Waltham, MA, USA) equipped with a Rtx-5MS on-column auto injector and a fused silica capillary column (DB-5, 30 m × 0.25 mm ID, 0.25 µm film thickness). Operating conditions were as follows: helium as the carrier gas with a flow rate of 1.5 mL/min; column temperature 40 °C as initial temperature, then increasing at 10 °C/min to 280 °C; injector temperature, 250 °C; volume injected, 1 µL; split ratio, 33.3. MS were recorded in electron ionization (EI) mode, with energy of 70 eV. The ion source temperature was 200 °C; 4.00 min solvent cut time. The compounds were identified by comparison with the data held in the NIST 11 Library; data are reported as *m/z* ratio with the relative abundance of the respective peak. Melting points (uncorrected) were measured on a Fisher-Johns melting point apparatus. Reaction progress was monitored by means of thin-layer chromatography using silica gel 60 (Merck, Darmstadt, Germany). All reagents were purchased from either Merck or Sigma Aldrich (St. Louis, MO, USA) and used without further purification. Final purification of all products for analysis was carried out by recrystallization.

### 3.2. Synthesis

The Knorr pyrazole synthesis is the reaction of hydrazine or substituted hydrazine with 1,3-dicarbonyl compounds to provide the pyrazole or pyrazolone ring system. Dimethylformamide (DMF) and acetic acid (AcOH) were used in the conventional method for the purpose of comparing the influence of the solvent on yield and reaction time.

Method A (Reflux)
  (a) A mixture of 2-acetylcyclohexanone (**1**, 1 mmol), and hydrazines **2** (1.0 mmol) in DMF (10 mL) was heated under reflux for 6–8 h. The precipitated solid product was filtered, washed with ethanol (EtOH), dried and finally recrystallized from DMF.
  (b) 2-Acetylcyclohexanone (**1**, 1 mmol) and hydrazines **2** (1.0 mmol) in AcOH (10 mL) were stirred at 80 °C for the given times. After completion of the reaction, the mixture was cooled to room temperature and the precipitate was filtered and purified by recrystallization from EtOH.
Method B (MW)

A mixture of 2-acetylcyclohexanone (**1**, 1.0 mmol) and hidrazines **2** (1.0 mmol) was subjected to microwave irradiation (Scheme 1). The solid products were isolated by crystallization of the reaction mixture from EtOH and washed with a mixture of hexane/ethanol (7:3) to get the corresponding compounds. Hydrazines used to obtain compounds **3c**–**f** was applied as the corresponding hydrochloride.

*3-Methyl-4,5,6,7-tetrahydro-1H-indazole* (**3a**). Yellow oil. Yield 90%, IR (KBr) ν/cm^−1^ 3150 (–CH_3_), 2919, 2841 (CH_2_), 1601, 1440 (C=C arom.), 1319, 845, 822; ^1^H-NMR (400.1 MHz, CDCl_3_) δ 1.67–1.62 (m, 4H), 2.11 (s, 3H), 2.30 (t, 2H, *J* 6.0), 2.53 (t, 2H, *J* 5.6), 9.68 (s, 1H); ^13^C-NMR (100.6 MHz, CDCl_3_) δ 10.28, 19.94, 22.20, 23.0, 23.41, 112.62, 140.14, 1143.67. GC-MS *m/z* (rel. int. %): 136 (40), 108 (100), 94 (10). Preparation of compound **3a** and other derivatives with a yield of 77% using cyclic (alkyl) (amino) carbene (CAAC)-gold(I) catalysts, has been described previously [39], and the use of tungstate sulfuric acid, under solvent-free conditions has been reported to give a similar yield [40].

*3-Methyl-2-phenyl-4,5,6,7-tetrahydro-2H-indazole* (**3b**). Yellow oil. Yield 90%, IR (KBr) ν/cm^−1^ 3316, 3107 (–CH_3_), 2969, 2876 (–CH_2_–), 1750 (C=N), 1618, 1590, 1504, 1423 (C=C arom.). ^1^H-NMR (400.1 MHz, CDCl_3_) δ 1.74–1.63 (m, 4H), 2.01 (s, 3H), 2.28 (t, 2H, *J* 6.0), 2.54 (t, 2H, *J* 6.0), 7.25–7.18 (m, 5H); ^13^C-NMR (100.6 MHz, CDCl_3_) δ 10.60, 20.30, 22.78, 23.18, 23.21, 115.27, 124.30, 126.73, 128.73, 134.44, 139.90, 149.51. GC-MS *m/z* (rel. int. %): 212 (100), 197 (34), 184 (78), 118 (32), 77 (82), 51 (20). Compound **3b** and other derivatives have been previously prepared by the condensation reaction of 1,3-diones and hydrazines under layered zirconium sulfophenyl phosphonate catalysis, under solvent-free conditions [41]. The use of tungstate sulfuric acid at 80 °C under solvent-free conditions has been reported to give a yield of 80% [40].

*2-(4-Bromophenyl)-3-methyl-4,5,6,7-tetrahydro-2H-indazole* (**3c**). Brown solid. Yield 90%, IR (KBr) ν/cm^−1^ 3037 (–CH_3_), 2907, 2846 (–CH_2_–), 1679 (C=N), 1605, 1496, 1440 (C=C arom.), 1066 (C-Br), 835 (–C_6_H_4_-Br). ^1^H-NMR (400.1 MHz, CDCl_3_) δ 1.69 (m, 4H), 2.12 (s, 3H), 2.37 (m, 2H), 2.61 (m, 2H), 7.44–7.23 (m, 3H); ^13^C-NMR (100.6 MHz, CDCl_3_) δ 10.84, 20.41, 22.89, 23.32, 23.79, 115.91, 120.22, 120.22, 123.88, 125.69, 131.98, 134.38, 139.21, 150.21.GC-MS *m/z* (rel. int. %): 292 (90), 289 (40), 275 (20), 155 (68), 120 (42), 91 (30), 77 (40), 76 (100).

*4-(3-Methyl-4,5,6,7-tetrahydro-2H-indazol-2-yl)benzonitrile* (**3d**). White solid. Yield 37%, IR (KBr) ν/cm^−1^ 3021 (–CH_3_), 2926, 2876 (–CH_2_–), 1654 (C=N), 1617, 1608, 1508, 1418 (C=C aromt). ^1^H-NMR (400.1 MHz, CD_3_OD) δ 1.77–1.65 (m, 4H), 2.26 (s, 3H), 2.46 (t, 2H, *J* 5.8), 2.64 (t, 2H, *J* 5.8), 7.63 (d, 2H, *J* 8.00), 7.82 (d, 2H, *J* 8.0); ^13^C-NMR (100.6 MHz, CD_3_OD) δ 13.87, 26.19, 26.57, 26.84, 27.65, 113.62, 120.78, 125.87, 127.89, 137.03, 139.55, 147.23, 155.13. GC-MS *m/z* (rel. int. %): (50), 222 (24), 209 (48), 143 (32), 102 (100), 77 (12).

*4-(3-Methyl-4,5,6,7-tetrahydro-2H-indazol-2-yl)benzoic acid* (**3e**). Orange crystal. Yield 40%, IR (KBr) ν/cm^−1^ 3437 (–COOH), 3162 (–CH_3_), 2934, 2841, 2780 (–CH_2_–), 1693 (C=N), 1606, 1573, 1517, 1490 (C=C arom.), 1427 (–OH), 1277 (C-O). ^1^H-NMR (400.1 MHz, DMSO *d_6_*) δ 1.74–1.68 (m, 4H), 2.26 (s, 3H), 2.42 (t, 2H, *J* 5.8), 2.57 (t, 2H, *J* 5.8), 7.63 (d, 1H, *J* 8.9), 8.02 (d, 1H, *J* 8.9), 12.98 (s, 1H); ^13^C-NMR (100.6 MHz, DMSO *d_6_*) δ 10.92, 19.87, 22.79, 22.85, 22.93, 116.03, 122.73, 128.15, 130.24, 134.48, 143.34, 149.70, 166.67. GC-MS *m/z* (rel. int. %): 256 (100), 239 (10), 228 (10), 139 (10), 123 (50).

*2-(4-Fluorophenyl)-3-methyl-4,5,6,7-tetrahydro-2H-indazole* (**3f**). Brown solid. Yield 98%, IR (KBr) ν/cm^−1^ 3030 (–CH_3_), 2893, 2846 (–CH_2_–), 1673 (C=N), 1605, 1496, 1440 (C=C arom.), 1060 (C-F), 850 (–C_6_H_4_-F). ^1^H-NMR (400.1 MHz, CDCl_3_) δ 1.69 (m, 4H), 2.01 (s, 3H), 2.37 (m, 2H), 2.79 (m, 2H), 7.01–7.16 (m, 4H); ^13^C-NMR (100.6 MHz, CDCl_3_) δ 9.40, 18.46, 20.72, 21.17, 21.42, 116.12, 125.75, 127.14, 129.98, 139.74, 142.59, 160.55. GC-MS *m/z* (rel. int. %): 230 (70), 214 (40), 217 (20), 155 (68), 120 (40), 91 (30), 77 (30), 76 (100).

### 3.3. X-ray Crystallography

The X-ray data of **3e** was collected at 297 (2) K with a Bruker CCD diffractometer using MoKα radiation [42]. The structure was solved by direct methods and refined with the full-matrix least-squares method on F^2^ with the use of SHELX-97 program package [43]. H-atoms of water molecules were found in difference Fourier maps and refined freely. Other H-atoms were placed in calculated positions. The isotopic displacement parameters were calculated as follows: *U*_iso_(H) = 1.2 *U*_eq_(O) and *U*_iso_(H) = *k U*_eq_(C), where *k* = 1.5 and 1.2.

### 3.4. Biological Activity

All compounds were evaluated as antioxidant agents. Each sample was dissolved in dimethylsulfoxide (DMSO) to make a solution of 1 mg/mL concentration, and then diluted to prepare the series concentrations for antioxidant assays. In all assays the final concentration of DMSO was inferior to 5%. Reference chemicals were used for comparison in all assays.

#### 3.4.1. Measurement of DPPH Radical Scavenging Activity

**Scheme 2:**
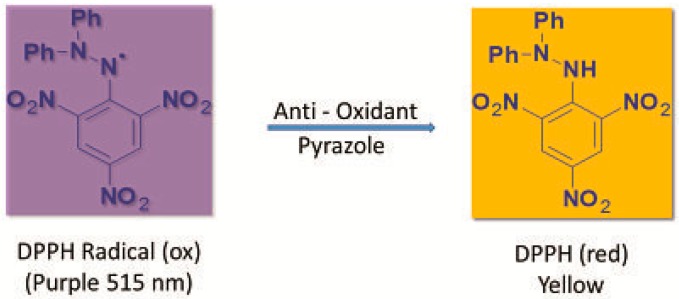
Visual change in the coloration in DPPH assay.

1,1-Diphenyl-2-picrylhydrazyl (DPPH) is a well-known radical which reacts with different antioxidant compounds where by its deep violet color in methanol solution changes to yellow (Scheme 2), the hydrogen atom or electron donation ability of the compounds was measured from the bleaching of the purple colored methanol solution of DPPH.

The scavenging activities of the compounds were estimated using DPPH as the free radical model according to the method previously described and adapted [44,45]. Briefly, an aliquot of 1 mL of the tested compound (10, 50 and 100 μg/mL) and control (2% DMSO final), respectively, were mixed with 2 mL of a methanolic solution of DPPH (0.5 mg/mL). The mixture was shaken vigorously and left to stand at room temperature for 5 min, in the absence of light. The mixture was measured spectrophotometrically at 515 nm. The free radical scavenging activity was calculated as percentage of DPPH decoloration using the following equation:

%scavenging DPPH free radical = 100 × (1 − AE/AD)

where AE, is the absorbance of the solution after adding the extract and AD is the absorbance of the blank DPPH solution. Ascorbic acid was used as reference compounds, with IC_50_ value of 1 µg/mL.

#### 3.4.2. Measurement of ABTS Radical Scavenging Activity

**Scheme 3:**
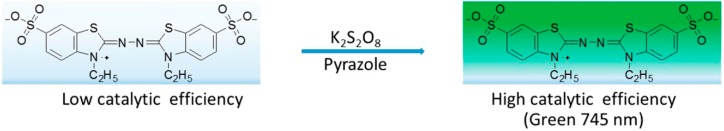
Visual change of coloration in ABTS assay.

The 2,2′-azino-bis(3-ethylbenzothiazoline-6-sulfonic acid (ABTS) radical scavenging assay is a rapid and efficient method, based on the ability of the hydrogen donating antioxidants to scavenge the long-life radical cation ABTS+. In this method, the preformed radical monocation of ABTS is generated by oxidation of ABTS with potassium persulfate (a blue chromogen) and is reduced in the presence of such hydrogen donating antioxidants (Scheme 3).

The ABTS assay was performed according to a published protocol [46]. The stock solution was prepared by mixing equal volumes of 7 mM ABTS solution and 2.45 mM potassium persulfate solution followed by incubation for 12 h at room temperature in the dark to yield a dark-colored solution containing ABTS•+ radicals. A working solution was freshly prepared before each assay by diluting the stock solution by mixing of stock solution to 50% methanol for an initial absorbance of about 0.700 (±0.02) at 745 nm, with temperature control set at 30 °C. Free radical scavenging activity was assessed by mixing 300 μL of different compounds (25–250 μg/mL in respective solvents) with 3.0 mL of ABTS working standard. The decrease in absorbance was measured exactly 1 minute after mixing the solution, the final absorbance was noted up to 6 min. Data for each assay was recorded in triplicate. Ascorbic acid, with an IC_50_ value of 35 µg/mL, was used as positive control. The scavenging activity was estimated based on the percentage of ABTS radicals scavenged by the following formula:

%scavenging = [(A0 − As)/A0] × 100

where A0 is absorption of control, As is absorption of tested compound solution.

## 4. Conclusions

In summary, in the present investigation we have described a simple and green method for the synthesis of a novel series of substituted tetrahydroindazole derivatives. The structures of these compounds were confirmed by spectral analysis and their antioxidant activity was evaluated. The results reveal that most of the synthesized tetrahydroindazoles can be considered as a scaffold for the development of novel and effective antioxidant agents. The antioxidant activity shown by the compounds obtained is totally dependent on the concentration and the type of substituent having the tetrahydroindazole ring. The use of other substituents can lead to improvements in antioxidant activity reported. Other tests to evaluate antioxidant activity can complement the results obtained and also provide information related to the mechanism of action displayed by these compounds.

The synthesis of tetrahydroindazole compounds via the microwave-assisted method of reaction is clean and offers shorter reaction times, with mild reaction conditions, is eco-friendly, produces excellent yields as compared to conventional methods, and reduces the use of volatile organic compounds. Finally, it is aligned with the principles of green chemistry. The uses of other conditions were evaluated to achieve improvements in the yields of derivatives **3d** and **3c**. Additional work is in progress in order to obtain different kinds of heterocycles using the same strategy.

## Figures and Tables

**Table 1:** Comparative study of conventional vs. microwave method.

Compound	R	Reflux		Microwave (MW)		M.p. °C
		DMF	Acid Acetic	300 W/150 °C		
		Time Reaction (h)/Yield (%)		Time Reaction (min)/Yield (%)		
**3a**	H	6/55	2/28	2	90	Oil
**3b**	C_6_H_5_	5/60	2.5/46	2	90	Oil
**3c**	4-BrC_6_H_4_	7/47	3/41	2	90	150–152
**3d**	4-CNC_6_H_4_	6/28	2/22	2	37	178–180
**3e**	4-CO_2_HC_6_H_4_	8/33	2/25	2	40	133–135
**3f**	4-FC_6_H_4_	7/61	3.5/43	2	98	118–120

**Table 2:** Temperature optimization for the synthesis of **3c** using MW at 300 W.

Entry	Reaction Temperature °C	Reaction Time (min)	Isolated Yield (%)
1	80	5	-
2	100	4	32
3	120	4	53
4	150	2	90
5	180	5	90

**Table 3:** Crystal data and details of the structure determination.

Compound	C_15_H_17_N_2_O_2_, H_2_O
Crystal shape, color	Polyhedron, yellow
Crystal size (mm)	0.32 × 0.12 × 0.10
Crystal system, Space group	Monoclinic, P2_1_/*n* (N°14)
a (Å)	10.704 (2)
b (Å)	10.106 (2)
c (Å)	13.735 (3)
β (°)	95.35 (3)
V (Å^3^)	1479.3 (5)
Z	4
Wavelength, Mo Kα (Å)	0.71073
T (K)	297 (2)
F(000)	580
*θ*-range (°)	2.50 < *θ* < 25.0
*hkl*-range	−12:12, −12:11, −16:16
μ (mm^−1^)	0.086
Reflections collected/*R*_int_/*R*_σ_	10,360/0.099/0.0909
Reflections unique/ parameters	2588/ 412
*R_1_*, *wR_2_* [*F*^2^ > 2σ (*F*^2^)]	0.0586, 0.0927
Goodness-of-Fit on *F^2^* (GooF = S)	1.106
Residual electron density **∆**ρ_max_/**∆**ρ_min_ (e Å^−3^)	0.15/−0.11

**Table 4:** Decoloration of DPPH solution by the synthesized compounds in comparison with ascorbic acid. Values are the average of triplicate experiments.

Compound/(µg/mL)	100	50	10	IC_50_
**3a**	13.51 ± 1.5	8.10 ± 0.6	3.78 ± 0.3	>100
**3b**	29.19 ± 4.3	17.84 ± 3.4	11.09 ± 1.7	>100
**3c**	31.35 ± 2.6	16.49 ± 2.2	8.92 ± 1.0	>100
**3d**	25.89 ± 5.3	13.45 ± 2.8	3.05 ± 0.8	>100
**3e**	47.72 ± 4.4	21.32 ± 2.1	7.87 ± 1.2	>100
**3f**	85.41 ± 12.6	55.14 ± 9.7	19.46 ± 6.7	42.35 ± 4.3
**Ascorbic acid**	-	-	-	1 ± 0.3

**Table 5:** ABTS radical scavenging activity of the synthesized compounds measured at 745 nm as compared to standard ascorbic acid. Values are the average of triplicate experiments.

Compound/(µg/mL)	100	75	50	25	IC_50_
**3a**	56.47 ± 12.2	35 ± 8.7	28.62 ± 5.3	0	87.94 ± 15.8
**3b**	52.14 ± 10.5	37.23 ± 11.6	12.04 ± 8.1	5.08 ± 0.8	92.15 ± 18.3
**3c**	46.88 ± 9.7	27.38 ± 9.9	19.67 ± 7.4	9.19 ± 1.7	>100
**3d**	21.34 ± 10.3	17.65 ± 8.7	12.10 ± 5.5	5.55 ± 2.4	>100
**3e**	49.24 ± 15.3	31.09 ± 9.0	3.87 ± 1.6	0	>100
**3f**	93.12 ± 17.5	88.37 ± 10.3	83.79 ± 8.5	53.65 ± 3.5	17.54 ± 4.8
**Ascorbic Acid**	-	-	-	-	35 ± 2.7
